# High sensitive troponine T as a biomarker and determinant factor of myocardial dysfunction in sepsis

**DOI:** 10.1186/2197-425X-3-S1-A303

**Published:** 2015-10-01

**Authors:** J Gonzalez Londoño, C Murcia Gubianas, N Garcia Febrer, C Lorencio, A Ochagavía, JM Sirvent

**Affiliations:** University Hospital Dr Josep Trueta, Intensive Care Unit, Girona, Spain; Parc Taulí Hospital, Intensive Care Unit, Sabadell, Spain

## Objectives

To determine whether the plasma concentration of high sensitive Troponin T (TnThs) correlates with the initial echocardiographic ejection fraction (EF) in patients with sepsis. Analyze whether the TnThs acts as a biomarker and independent determinant factor of myocardial dysfunction in these patients.

## Methods

We performed a prospective observational study with a cohort of 60 septic patients admitted to the ICU. Demographic data, severity scales, organ failure and doses of noradrenalin were registered. TnThs analysis was performed at baseline and in the first 24 hours a transthoracic echocardiography was performed to assess the ejection fraction (Simpson), once a correct resuscitation had been performed. Patients were monitored until hospital discharge. The statistical analysis was aimed to show whether biomarker TnThs acts as an early myocardial dysfunction biomarker in patients with sepsis and if it is an independent determinant factor of myocardial dysfunction. A bivariate analysis was performed using non-parametric tests and multiple logistic regression with the aim of obtaining a model adjusted to severity. Statistical significance if p < 0.05.

## Results

Of the 60 studied patients, 65% were men, with a mean age 63.9 ± 13.2 years. APACHE II was 16.8 ± 7.5 points, SAPS II 38.3 ± 15.7 points and an initial SOFA of 6.8 ± 2.8 points. Mortality to 28 days was 23.3%. The most frequent source of sepsis was abdominal (38.3%), followed by respiratory (23.3%). 36.7% of patients had initial myocardial dysfunction (EF < 50%) and TnThs values were positive (> 15 ng / L) in 76.6%.The correlation between the quantitative levels of initial TnThs and doses of noradrenaline was positive and statistically significant, with p = 0.0001. There was also a significant correlation, in this case negative, between the quantitative levels of initial TnThs and the initial EF (p = 0.04). The multiple logistic regression model adjusted for APACHE II showed that the TnThs [OR: 1.027 (95% CI = 1.000 to 1.007); p = 0.04] was an independent determinant of myocardial dysfunction in this model.

## Conclusions

The TnThs is a suitable biomarker for detecting myocardial dysfunction and correlates well with the initial EF and doses of noradrenalin. In a multiple logistic regression model adjusted by severity, TnThs behaves as an independent determinant factor of myocardial dysfunction in patients with sepsis.

